# Underdetermined Blind Source Separation with Variational Mode Decomposition for Compound Roller Bearing Fault Signals

**DOI:** 10.3390/s16060897

**Published:** 2016-06-16

**Authors:** Gang Tang, Ganggang Luo, Weihua Zhang, Caijin Yang, Huaqing Wang

**Affiliations:** 1 College of Mechanical and Electrical Engineering, Beijing University of Chemical Technology, Beijing 100029, China; tanggang@mail.buct.edu.cn (G.T.); luo93_buct@hotmail.com (G.L.); 2 Traction Power State Key Laboratory, Southwest Jiaotong University, Chengdu 610031, China; tpl@swjtu.edu.cn (W.Z.); ycj78_2012@163.com (C.Y.)

**Keywords:** roller bearing, fault diagnosis, variational mode decomposition, independent component analysis

## Abstract

In the condition monitoring of roller bearings, the measured signals are often compounded due to the unknown multi-vibration sources and complex transfer paths. Moreover, the sensors are limited in particular locations and numbers. Thus, this is a problem of underdetermined blind source separation for the vibration sources estimation, which makes it difficult to extract fault features exactly by ordinary methods in running tests. To improve the effectiveness of compound fault diagnosis in roller bearings, the present paper proposes a new method to solve the underdetermined problem and to extract fault features based on variational mode decomposition. In order to surmount the shortcomings of inadequate signals collected through limited sensors, a vibration signal is firstly decomposed into a number of band-limited intrinsic mode functions by variational mode decomposition. Then, the demodulated signal with the Hilbert transform of these multi-channel functions is used as the input matrix for independent component analysis. Finally, the compound faults are separated effectively by carrying out independent component analysis, which enables the fault features to be extracted more easily and identified more clearly. Experimental results validate the effectiveness of the proposed method in compound fault separation, and a comparison experiment shows that the proposed method has higher adaptability and practicability in separating strong noise signals than the commonly-used ensemble empirical mode decomposition method.

## 1. Introduction

Roller bearings are important components in rotating machines, which are widely used in industrial applications. However, roller bearings are also one of the most vulnerable components of these machines [1]. According to the statistics, 30% of all mechanical faults are caused by malfunctioning roller bearings; hence, bearing faults may cause huge economic losses [2]. Therefore, detecting roller bearing faults in a timely and accurate manner has become increasingly important.

In general, vibration signals are used to detect roller bearing faults because these signals provide useful information about different fault features. However, as a typical non-stationary signal, vibration signals of roller bearings are usually interfered by a large amount of noise in practical tests. In addition, most bearing faults are often compounded by different fault features, namely the outer-race defect, inner-race defect and roller defect. So far, many studies have been conducted on the fault diagnosis of roller bearings.

The fast Fourier transform (FFT) and other techniques based on it are widely used to extract the features of a bearing [3,4]. However, in the case of nonstationary signals, it is difficult to use FFT to interpret and extract the features in the time domain. To solve this problem, a spectrogram method based on time-frequency representations and a wavelet method-based time-scale analysis have been proposed [5,6]. Nevertheless, the accuracy of these methods depends on the data length, and they have poor effectiveness in extracting compound faults.

To overcome these problems and improve the identification of compound faults, Herault proposed a method called blind source separation (BSS) to separate compound faults. BSS is the recovery of the original source signals from mixed signals when only a priori information is available [7]. Independent component analysis (ICA) is one of the main approaches to realizing BSS without the need for prior information about mixed signals [8]. However, ICA requires that the number of sensors be equal to or greater than the number of original source signals. In fact, because of the particularity of mechanical system, sensors are limited in particular locations and numbers so that it is a problem of underdetermined blind source separation, for which is difficult to extract fault features using conventional methods. To solve this problem, many researchers have applied decomposition methods to mixed signals, such as wavelet transform [9], local mean decomposition [10] and empirical mode decomposition (EMD) [11]. EMD can self-adaptively decompose nonstationary signals under the simple assumption that signals consist of different intrinsic oscillation modes, which can be used to extract the intrinsic mode functions (IMFs). EMD has been successfully applied to numerous investigation fields, such as acoustic, biological, ocean, earth-quake, climate, fault diagnosis, *etc.* [12]. In general, the number of IMFs obtained from EMD is considerably greater than the number of independent components in the signal [13]. Therefore, the IMFs can be used as the input matrix for ICA, and some research has been conducted on extracting fault features by combining EMD with ICA [14,15,16,17]. However, the major drawback of EMD is mode mixing [18,19], which is caused by signal intermittence. To solve this problem, a noise-assisted method called ensemble EMD (EEMD) was proposed by Wu and Huang [20]. EEMD can not only maintain the characteristics of EMD, but also can eliminate mode mixing effects by adding white noise, which has the statistical characteristic of the uniform distribution of frequency into the original signal. However, EEMD gives rise to a huge computational load and depends on the noise and sampling rate, which inhibit its application to noisy industrial environments.

A recent novel technique called variational mode decomposition (VMD) [21] has provided new insight into the extraction of fault features. VMD can non-recursively decompose a multi-component signal into a number of ensembles of band-limited modes. However, EMD decomposes data into IMFs without band-limited properties. For the vibration signals of roller bearings, the frequency is exactly band limited. The band-limited a priori information guarantees high effectiveness in noisy environments. In addition, unlike EMD, which models the individual modes as signals with explicit IMFs, VMD begins with the variational energy minimization of band-limited intrinsic mode functions (BLIMFs). The BLIMFs are extracted concurrently instead of recursively, thus producing high efficiency. In a recent research work, Mohanty preformed the signal analysis of bearing faults using VMD [22]. He illustrated that VMD can remove the exponentially decaying DC offset, and he evaluated the performance of VMD compared to that of EMD [23]. However, its application in the area of compound fault diagnosis has been rarely reported.

In order to separate the mixed vibration signal and to extract the fault characteristic frequency effectively and practicably, a new method based on VMD is proposed in this paper. First, a vibration signal is decomposed by VMD to solve the underdetermined problem. Second, the demodulated signal of BLIMFs with the Hilbert transform is used as the input matrix for ICA. Finally, the compound faults can be separated and identified. In addition, a comparison is made to investigate the effectiveness of the proposed method compared to that of the EEMD method [24]. The rest of the paper is organized as follows. Underdetermined blind source separation is discussed in Section 2. In Section 3, the VMD algorithm is reviewed. Section 4 presents the ICA algorithm. The proposed method can be seen in Section 5. Section 6 contains a description of the experimental test, the algorithm application and a comparison of the proposed method and EEMD. The paper is concluded in Section 7.

## 2. Underdetermined Blind Source Separation

Blind source separation (BSS), also called blind signal separation, is the process of recovering each source signal only from the observed signal based on the statistical characteristics of the input signal without the parameters of the source signal and the transmission channel. The purpose of BSS is to confirm the parameters of the separation process and to obtain the estimation of the source signal based on the observed signal. It can be represented as the following formula:

(1) $$x_{i}\left(t\right)=\overset{n}{\underset{j=i}{\sum }}\alpha_{ij}s_{j}\left(t\right)+n_{j}\left(t\right)\;i=1,2,\cdots ,n\;t=1,2,\cdots ,T$$

where $x_{i}\left(t\right)$ is the observed signal, $s_{i}\left(t\right)$ is the source signal, $\alpha_{ij}$ is the mixing matrix, $n_{i}\left(t\right)$ is the noise signal and T is the sampling data. Formula (1) can be described as a matrix form:

(2) X(t)=AS(t)+N(t)

where $S\left(t\right)={\left[s_{1}\left(t\right),s_{2}\left(t\right),\cdots ,s_{m}\left(t\right)\right]}^{T}$ is m original signals, A is the n×m hybrid matrix and $X\left(t\right)={\left[x_{1}\left(t\right),\;x_{2}\left(t\right),\cdots ,\;x_{n}\left(t\right)\right]}^{T}$ is the n-d observed signal. In general, X is considered as the combination of original signal S and hybrid matrix A (X=AS). Under the condition that both S and A are unknown, demixing matrix B is acquired to make sure that Y obtained by X and B is the best estimation of S.

Because the prior knowledge of the original signal and the hybrid matrix are unknown, some basic assumptions must be stipulated as follows:

(1) source signals are statistically independent of each other;
(2) hybrid matrix A is a matrix with full column rank;
(3) noise signals are statistically independent of each other and are irrelevant to the original signals.
(4) The number of source signals (N) is less than or equal to the number of observed signals (M).

In practice, the position and quantity of sensors are limitative, which usually causes the number of sensors to be less than the number of source signals. This condition that M<N is called underdetermined blind source separation.

Compared to BSS, underdetermined blind source separation is a hot spot in the field of modern signal processing. This paper uses variational mode decomposition to separate the observed signal, which turns the underdetermined problem into a positive definite or super positive definite problem. Then, independent component analysis is used to solve this problem and to obtain the source signals.

## 3. Variational Mode Decomposition 

VMD is a newly-developed time-frequency analysis method for adaptive signal decomposition, which can decompose a multicomponent signal into a number of band-limited IMFs (BLIMFs) through an iteration solving process of a special variational model. The mathematical model of VMD is as follows.

An original signal x(t) can be decomposed into a limited number of sub-signals $u_{k}$ that have different center frequencies $\omega_{k}$ and limited bandwidths.

First, the one-sided spectrum of $u_{k}$ is obtained by the Hilbert transform:

(3) $$\left(\delta \left(t\right)+\frac{j}{\pi t}\right)*u_{k}\left(t\right)$$

where δ(t) is the Dirichlet function and * is the symbol of the convolution operation.

Then, the spectrum of each mode is transferred into the baseband by frequency mixing:

(4) $$\left[\left(\delta \left(t\right)+\frac{j}{\pi t}\right)*u_{k}\left(t\right)\right]e^{-jw_{k}t}$$

Next, the bandwidth of each mode can be estimated by calculating the $L_{2}$-norm of the demodulated signal shown in Formula (4).

Finally, VMD is built as a constrained variational model [21]:

(5) $$\underset{\left\{u_{k}\right\},\left\{\omega_{k}\right\}}{\min}\left\{\sum_{k}\|\partial_{t}\left[\left(\delta \left(t\right)+\frac{j}{\pi t}\right)*u_{k}\left(t\right)\right]e^{-jw_{k}t}{\|}_{2}^{2}\right\}\;s.t.\;\;\sum_{k}u_{k}=x$$

where $\left\{u_{k}\right\}=\left\{u_{1},\cdots \cdots \cdots ,u_{k}\right\}$ and $\left\{\omega_{k}\right\}=\left\{\omega_{1},\cdots \cdots \cdots ,\omega_{k}\right\}$ are a series of modes and their center frequencies, respectively.

To obtain the optimal solution of the above variational model, the quadratic penalty factor α and the Lagrange multiplier λ are imported into VMD. By turning the constrained problem into an under-constrained problem, the signal can be set using Equation (4):

(6) $$L\left(\left\{u_{k}\right\},\left\{\omega_{k}\right\},\lambda \right)=\alpha \underset{k}{\sum }\|\partial_{t}\left[\left(\delta \left(t\right)+\frac{j}{\pi t}\right)*u_{k}\left(t\right)\right]e^{-j\omega_{k}t}{\|}_{2}^{2}+\|x\left(t\right)-\underset{k}{\sum }u_{k}\left(t\right){\|}_{2}^{2}+\langle \lambda \left(t\right),x\left(t\right)-\underset{k}{\sum }u_{k}\left(t\right)\rangle$$

**Figure 1:**
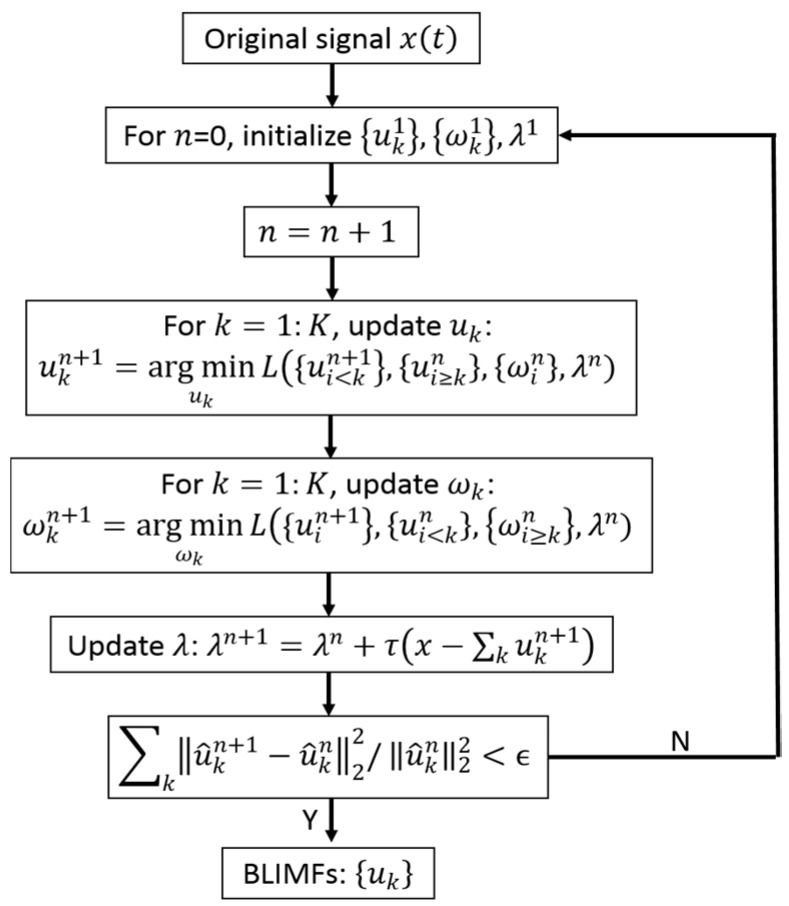
Flow diagram of the variational mode decomposition (VMD) algorithm.

Based on this, $\left\{u_{k}\right\},\;\left\{\omega_{k}\right\},\;\lambda$ are updated alternately by iterations using the alternative direction method of multipliers [25]. In addition, the BLIMFs, which are found in $\left\{u_{k}\right\}$, are decomposed from the original signal. The flow diagram of the VMD algorithm is shown in Figure 1.

## 4. Independent Component Analysis

Independent component analysis (ICA) [26,27,28] was developed in the late 1900s on digital signal processing. The main idea of ICA is to separate a set of independent sources from the original mixed signal. The mathematical model of ICA can be represented in terms of the vector-matrix notation as follows:

(7) $$X=AS=\overset{m}{\underset{i=1}{\sum }}a_{i}s_{i},\;i=1,2,\cdots ,m$$

where $X={\left[x_{1},x_{2},\cdots ,x_{n}\right]}^{T}$ is the random observed vector, $S={\left[s_{1},s_{2},\cdots ,s_{m}\right]}^{T}$ is the source signal and $A=\left[a_{1},a_{2},\cdots ,a_{m}\right]$ is the n×m mixing matrix.

In the model of ICA, it is a prerequisite not only for the source signals to be independent of each other, but also for the characteristics of the Gaussian distribution to be satisfied. In addition, for simplifying the model, the mixing matrix A is assumed to be a square matrix, which means m=n. To estimate the sources, the ICA algorithm considers a linear transformation according to the following equation:

(8) Y=WX=WAS=ZS

where Z=WA, W is one of the best transformation matrices and Y is the estimation of the independent component $s_{i}$.

To improve the efficiency and stability of the algorithm, this paper applies the FastICA algorithm, which is based on the fixed-point algorithm and can process a large number of sampling points of the observed vector X in batches using the Newton iteration algorithm. The process of the FastICA algorithm is as follows.

Step 1.

Remove the mean of the observed signal X:

(9) X=X−E{X}

Step 2.

Whiten the observed signal X:

(10) $$\overset{ˇ}{X}=ED^{-1/2}E^{T}X$$

where E is the orthogonal matrix of the eigenvectors of the covariance matrix $E\left\{XX^{T}\right\}$ of the observed signal X, $D=diag\left(d_{1},\;d_{2},\;\cdots \;,\;d_{n}\right)$ is the diagonal matrix and $d_{i}$ is the eigenvalue of $E\left\{XX^{T}\right\}$.

Step 3.

Choose an initial vector $W_{0}$ randomly with k=0.

Step 4.

Update $W_{k+1}$:

(11) $$W_{k+1}=E\left\{Xg\left(w_{k}^{T}\right)\right\}-E\left\{g^{,}\left(w_{k}^{T}X\right)W_{k}\right\}$$

Step 5.

Normalize $w_{k+1}$:

(12) $$W_{k+1}=W_{k+1}/\|W_{k+1}\|$$

Step 6.

If $\left|W_{k+1}-W_{k}\right|>\varepsilon$, which means that the solution did not converge, go back to Step 4. Otherwise, calculate one independent component Y=WX≈S.

## 5. Proposed Method

Vibration signal analysis is usually used for condition monitoring and fault diagnosis. However, because the fault in roller bearings is a compound fault caused by material fatigue and poor lubrication among the outer-race, inner-race and rollers, it is difficult to analyze and detect the fault characteristics in the spectrum. In addition, the noise environment is one of the main reasons for the separation of the compound fault from the vibration signal.

**Figure 2:**
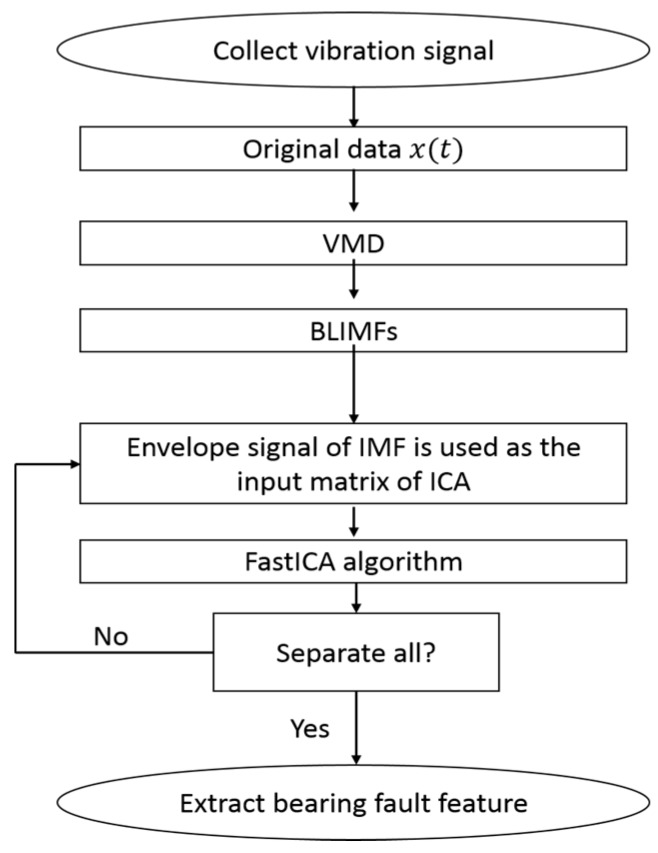
Detailed experimental scheme. BLIMF, band-limited intrinsic mode function; IMF, intrinsic mode function.

VMD is a recent novel technique that has provided new insight into the extraction of fault features. VMD can non-recursively decompose a multicomponent signal and can provide great stability in a noisy environment. To leverage these advantages, a new method based on VMD is proposed in this paper. To verify the efficiency of the proposed method, several experiments on bearing faults are performed. The detailed experimental scheme is shown in Figure 2. First, a signal channel vibration x(t), which is a compound fault of a bearing outer-race and a roller, is collected by the acceleration sensor vertically fixed on the bearing seat. Second, the collected vibration signal x(t) is decomposed into BLIMFs using the VMD method to obtain six multichannel signals. Third, the demodulated signal of BLIMFs with the Hilbert transform is used as the input matrix for ICA to separate the bearing compound fault. Lastly, the bearing fault features of the equipment are extracted by comparing the fault characteristic frequencies in the spectra with the theoretical characteristic frequencies of a roller bearing.

## 6. Experimental Results

### 6.1. Simulation

**Figure 3:**
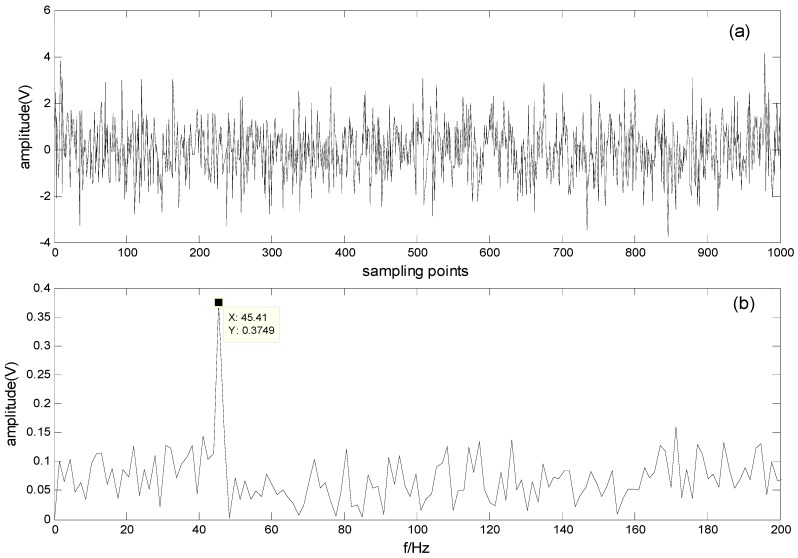
Simulated signal and its spectrum: (**a**) Waveform of the simulated signal; (**b**) Spectrum of the simulated signal.

A simulation is performed to verify the effectiveness of the proposed method. To simulate the compound fault signal for a roller bearing, three source signals, expressed in Equation (13), are randomly mixed according to Equation (14). The fault characteristic frequencies of the mixed signal are 45 and 120 Hz.

(13) $$\{\begin{array}{c} s_{1}=0.3\cos\left(90\pi t+0.5sin30\pi t\right)\\ s_{2}=0.2sin240\pi t\\ s_{3}=randn\left(1,N\right) \end{array}$$

(14) $$X=AS=A{\left[s_{1},s_{2},s_{3}\right]}^{T}$$

where A is a random mixing matrix. The mixed signal in the time and frequency domains is shown in Figure 3. As can be seen from Figure 3b, one of the fault characteristic frequencies (120 Hz) is difficult to spot in the spectrum.

**Figure 4:**
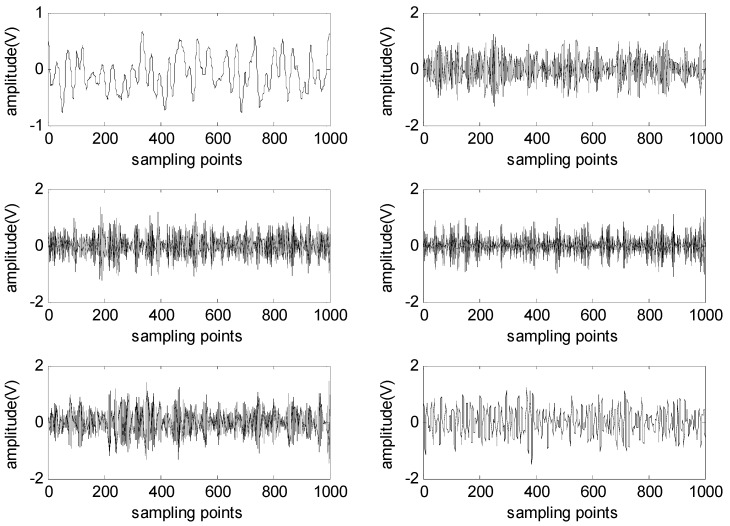
BLIMFs decomposed by the VMD method.

Then, according to the detailed experimental scheme shown in Figure 2, the mixed signal is first decomposed into six BLIMFs by the VMD method. The six BLIMFs are shown in Figure 4.

**Figure 5:**
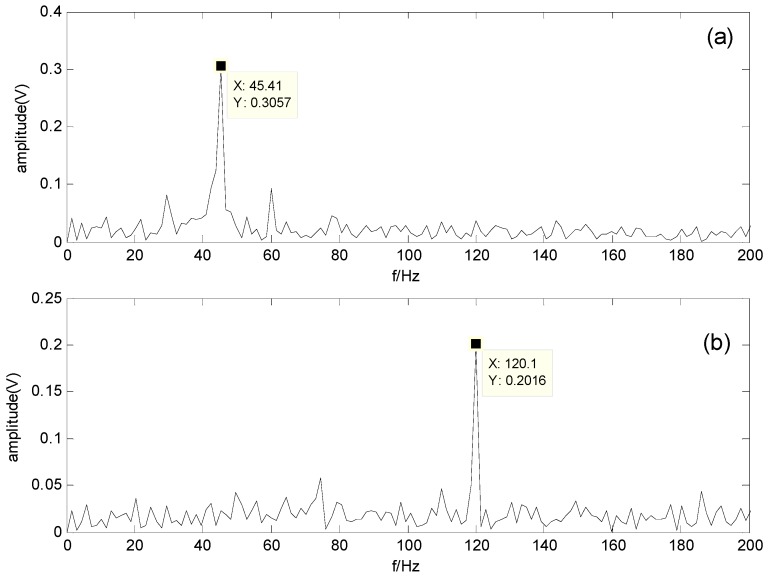
Spectrum of the signals separated by the proposed method: (**a**) Spectrum of IC1; (**b**) Spectrum of IC2.

Then, these BLIMFs are used as the input matrix for the FastICA algorithm to separate the compound fault. The separation results are shown in Figure 5. The results indicate that two kinds of fault signals with fault characteristic frequencies of 45 and 120 Hz are mixed in the original signal. The proposed method can be used to effectively separate the compound fault signals and accurately extract the fault characteristic frequencies of the roller bearings.

### 6.2. Experimental Setup

**Figure 6:**
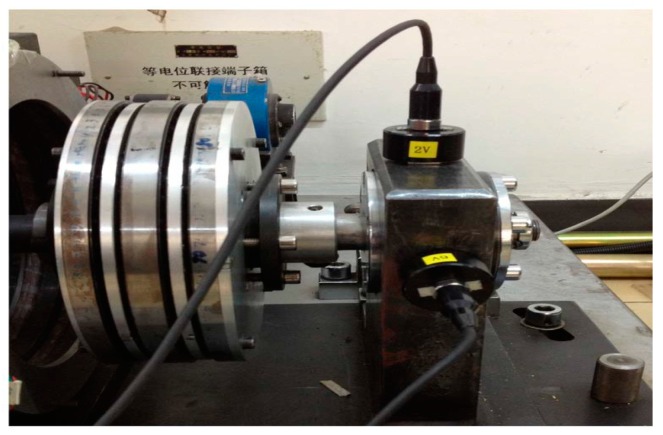
Experimental system and installation locations of the acceleration sensors.

To validate the efficiency of the proposed method in practical applications, an experimental system, including a rotating machine, a roller bearing and acceleration sensors for bearing fault diagnosis, is developed, as shown in Figure 6. The main acceleration sensors are mounted on the top and side of the bearing housing to measure the vibration signal of the 2-V channel. The compound fault of the bearing outer-race and rollers is considered as the research object. For the fault diagnosis test, an outer-race flaw (0.5 mm (width) × 0.15 mm (depth)) and a roller flaw (0.5 mm (width) × 0.15 mm (depth)) are artificially created using a wire-cutting machine. Additionally, the commercial code of the bearing is N205EM.

To fully analyze the signal features and acquire more comprehensive information on the fault diagnosis, a large sampling frequency (100 kHz) is chosen. The performance period of the experimental system is 10 s, and the rotating speeds of the rotating machine are set to 500, 900 and 1300 rpm.

### 6.3. Diagnosis by Traditional Envelope Spectrum Analysis

First, the theoretical fault features of the signals collected by the abovementioned experimental system are analyzed. The characteristic frequencies of the outer-race and rollers can be calculated by the following formulas.

The characteristic frequency of the outer-race defect $\left(f_{o}\right)$:

(15) $$f_{o}=\frac{Z}{2}\left(1-\frac{d}{D}cos\alpha \right)f_{r}$$

The characteristic frequency of the rollers’ defect $\left(f_{b}\right)$:

(16) $$f_{b}=\frac{D}{2d}\left[1-{\left(\frac{d}{D}cos\alpha \right)}^{2}\right]f_{r}$$

where Z is the number of rollers, d is the diameter of the rollers, D is the pitch diameter, α is the contact angle and $f_{r}$ is the rotating frequency of the rotating machine.

**C**alculations based on the above formulas give the characteristic frequencies of the roller defect and outer-race defect at 500, 900 and 1300 rpm, as listed in Table 1.

Then, the calculated characteristic frequencies are compared to the fault characteristic frequencies in the envelope spectrum to identify the cause of the defect. Because the original signal is a modulating signal, envelope detection should be used to process the signal in advance [29]. After this, the FFT-based Hilbert transform is applied to obtain the spectrum. For roller bearing diagnosis, the fault characteristic frequency does not always equal the calculated theoretic frequency. The reasons are as follows:

(1) The theoretical frequency is calculated based on the assumption of a pure rolling motion. However, in practice, some sliding motion may occur.
(2) In practice, some installation error will appear in the roller bearing.

Therefore, the calculated theoretical frequency should be regarded as an approximation only.

**Figure 7:**
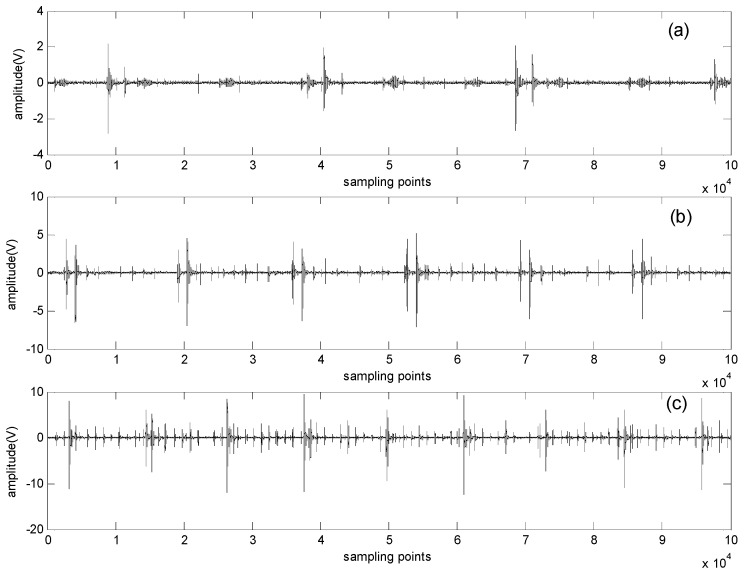
Original signal waveforms obtained at different rotating speeds: (**a**) 500 rpm; (**b**) 900 rpm; (**c**) 1300 rpm.

**Figure 8:**
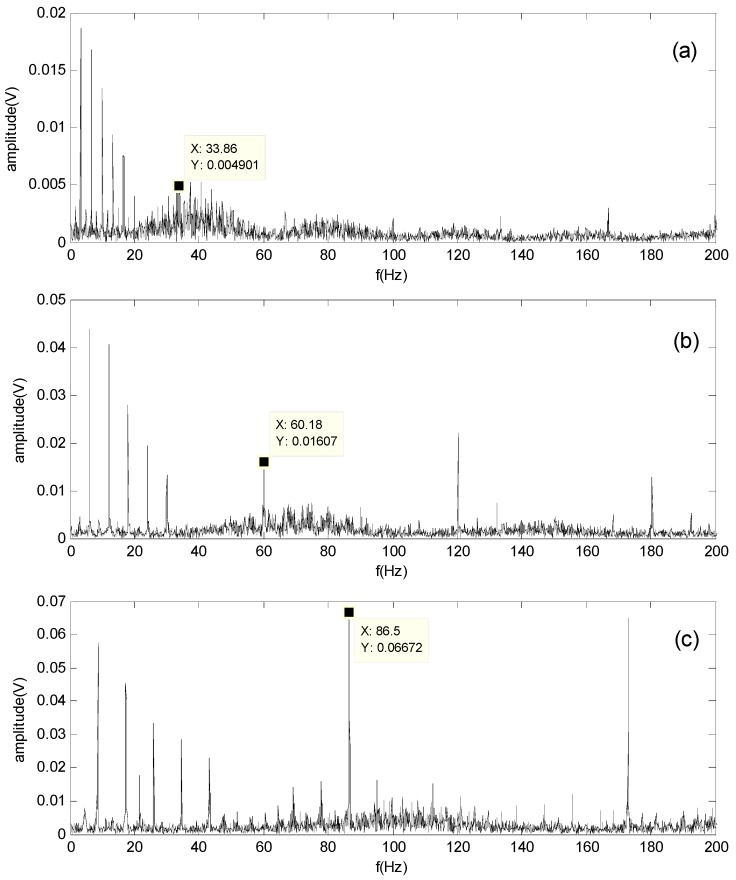
Envelope spectrum of the original signal at different rotating speeds: (**a**) 500 rpm; (**b**) 900 rpm; (**c**) 1300 rpm.

Figure 7 shows the original signals collected by the experimental system at different rotating speeds (500, 900 and 1300 rpm) in the time domain. Accordingly, the envelope spectrum obtained by the FFT-based Hilbert transform is shown in Figure 8.

As shown in Figure 8a, the theoretical characteristic frequency $f_{b}$ at 39.3 Hz is not clearly apparent, but the outer-race defect of the bearing can still be identified. Figure 8b,c also shows that $f_{o}$ is apparent, but the identification of $f_{b}$ is difficult according to the calculated characteristic frequency.

Because of the noise environment, the fault characteristic frequency of the roller defect is buried and difficult to identify in the spectrum, in which all types of bearing characteristic frequencies are located. Therefore, the compound faults in roller bearings cannot be completely detected by the traditional envelope analysis technique.

### 6.4. Diagnosis by the Proposed Method

**Figure 9:**
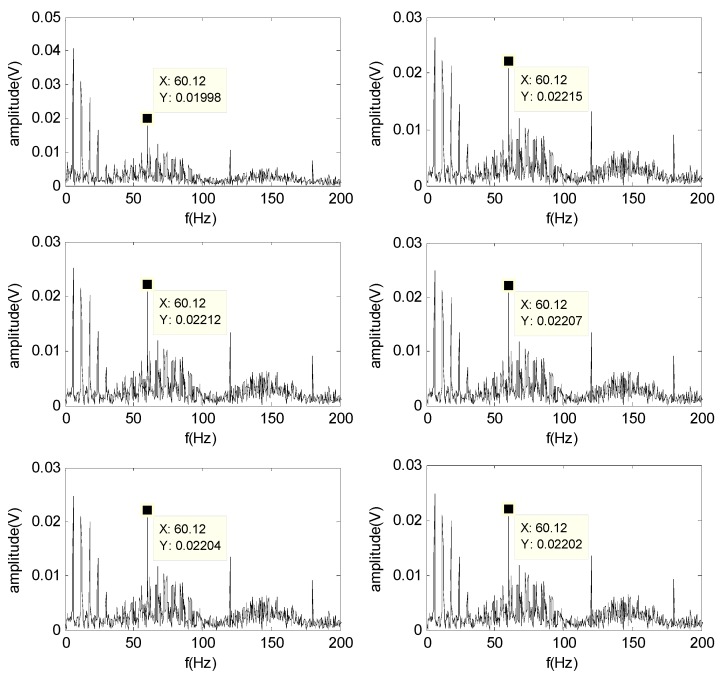
Envelope spectrum of BLIMF1–BLIMF6.

To solve the problem of underdetermined blind source separation, in which the observed signal numbers are less than the source numbers, the single-channel vibration signal collected by the acceleration sensor at 900 rpm is decomposed to BLIMFs by the VMD method to obtain multichannel signals. After executing the VMD method, six BLIMFs (the number can be manually set) are acquired. Figure 9 shows the envelope spectrum of BLIMF1-BLIMF6. The figure indicates that the fault characteristic frequency of the outer-race is apparent, but the features of other faults cannot be observed.

Then, to extract the fault signal that consists of outer-race and roller defect features, source signals are separated from the observed signal by utilizing the ICA technique. The diagnostic operational procedure is as follows.

**Figure 10:**
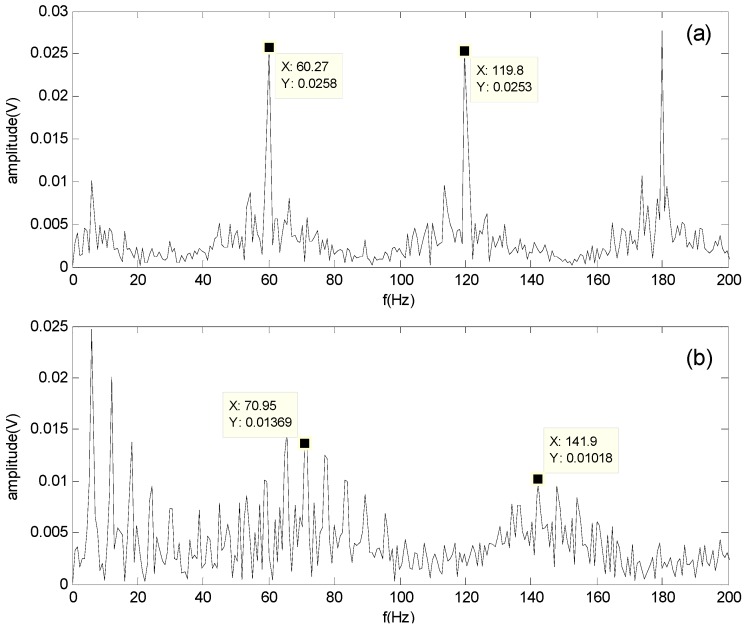
Spectrum of separated signals obtained by the proposed method at 900 rpm: (**a**) Spectrum of the outer-race defect; (**b**) Spectrum of the roller defect.

First, envelope analysis is carried out for BLIMF1–BLIMF6 through the Hilbert transform. Second, a 6×n matrix is acquired from the six BLIMFs, where n is the number of sampling points for the original signal. Third, the matrix is used as the input for the FastICA algorithm to obtain the independent component signal. Lastly, the condition of the bearings is diagnosed by comparing the spectrum of the separated signals with the calculated fault characteristic frequency. Figure 10 shows the results obtained by the proposed method at 900 rpm.

In Figure 10a, the frequency $f_{o}$ at 60.27 Hz is clearly apparent and very close to the calculated characteristic frequency of the outer-race defect at 59.8 Hz; therefore, the bearing outer-race defect can be identified. Similarly, in Figure 10b, the roller defect can be identified because the characteristic frequency $f_{b}$ at 70.95 Hz in the spectrum is close to the calculated characteristic frequency of the roller defect at 71.8 Hz. However, some confusing noise signals still exist in the spectrum of the roller defect; hence, the roller defect is more difficult to diagnose than the outer-race defect.

**Figure 11:**
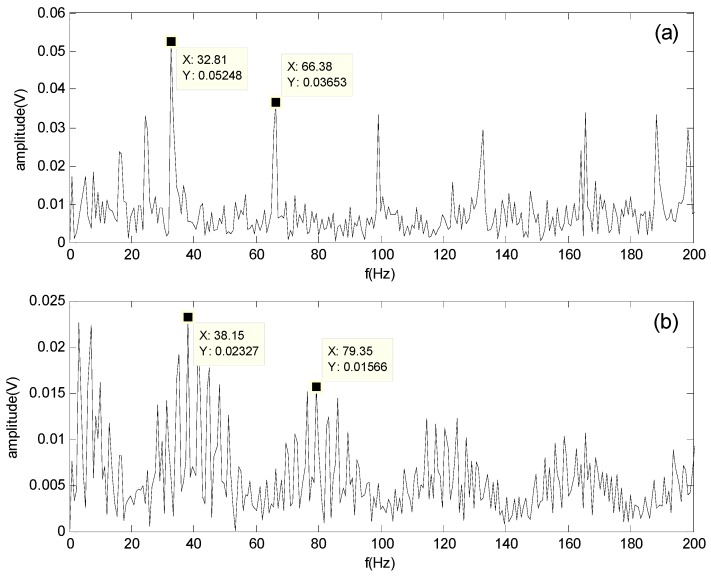
Spectrum of the separated signals obtained by the proposed method at 500 rpm: (**a**) Spectrum of the outer-race defect; (**b**) Spectrum of the roller defect.

**Figure 12:**
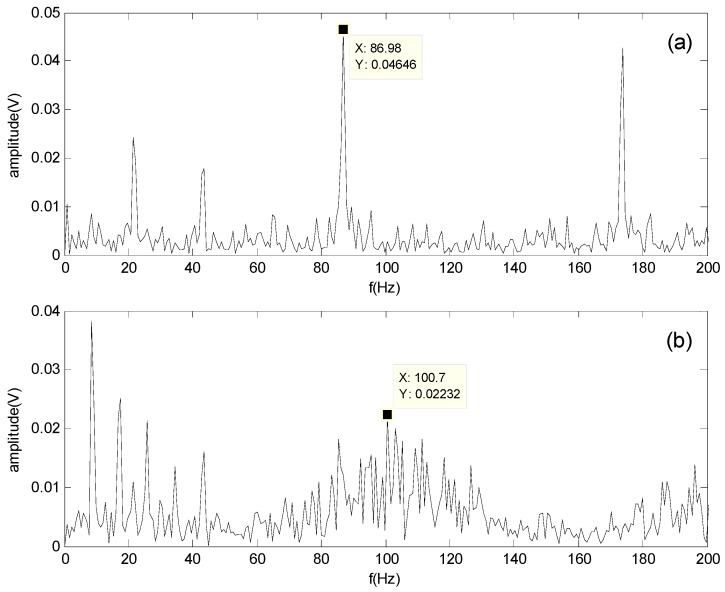
Spectrum of the separated signals obtained by the proposed method at 1300 rpm: (**a**) Spectrum of the outer-race defect; (**b**) Spectrum of the roller defect.

Figure 11 and Figure 12 show the spectrum of the bearing outer-race defect and roller defect at 500 and 1300 rpm, respectively. These figures indicate that the outer-race defect and roller defects can be clearly identified by the proposed method.

Based on the results, we can deduce that the compound fault contains the outer-race defect and roller defect in the experimental system shown in Figure 6. Hence, the proposed method is proven to be effective for compound fault diagnosis in roller bearings.

### 6.5. Comparison of the Proposed Method with the EEMD Method

**Figure 13:**
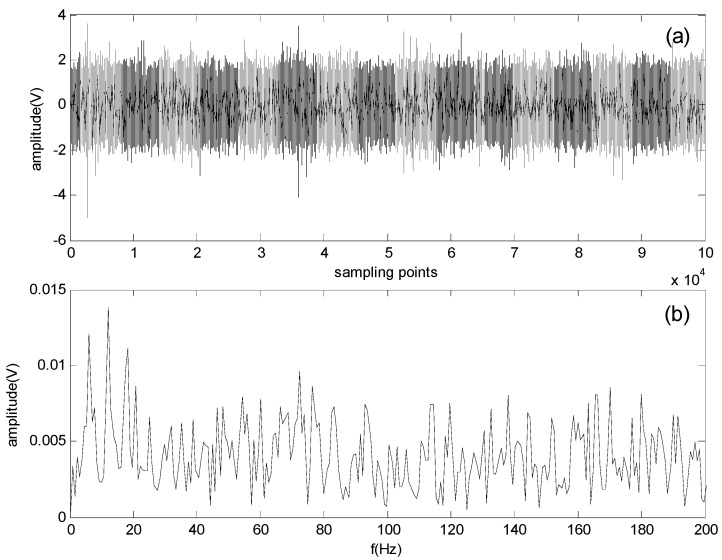
Noisy signal and its spectrum: (**a**) Waveform of the noisy signal; (**b**) Spectrum of the noisy signal.

EMD is very suitable for decomposing nonlinear and nonstationary time series, which can adaptively represent the local characteristics of a given signal. The main idea of EMD is to decompose time series data into a sum of oscillatory functions, namely, IMFs. However, the biggest drawback of EMD is mode mixing, which is defined as the formation of a signal IMF consisting of either signals with widely disparate scales or signals of similar scales residing in different IMF components. To overcome this problem, EEMD was proposed by adding a white noise signal to the original signal. This method, in which EEMD is used to decompose the original signal and ICA is used to extract the principal component, is found to be effective for compound fault diagnosis [24]. Because of its filtering process in the frequency domain, the proposed method gives better results than the EEMD-ICA method in the diagnosis of fault signals with low signal-to-noise ratios (SNRs). Original diagnosis signals with low SNRs at 900 rpm and their envelope spectra are shown in Figure 13 under the compound fault state of the outer race and rollers. The figure indicates that the original signal is very noisy and that the outer-race and roller fault frequencies are not visible in the spectrum.

**Figure 14:**
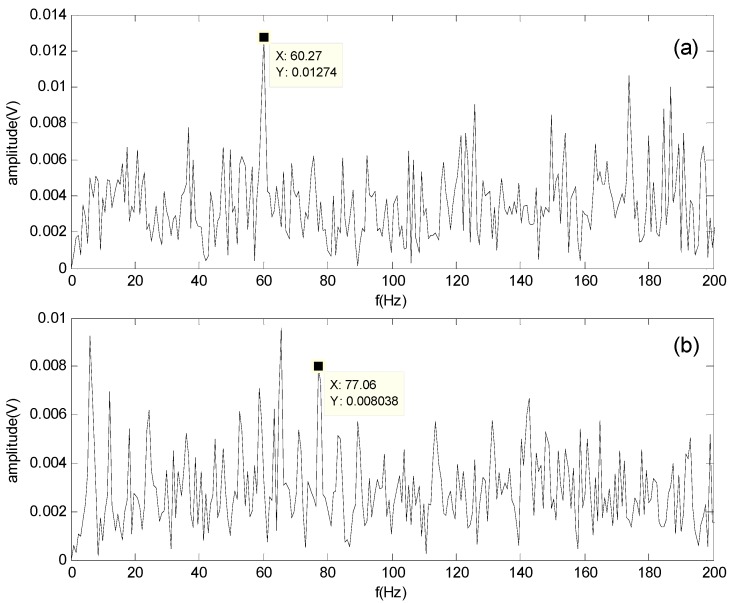
Spectrum of the separated noisy signals by ensemble empirical mode decomposition (EEMD): (**a**) Spectrum of the outer-race defect; (**b**) Spectrum of the roller defect.

**Figure 15:**
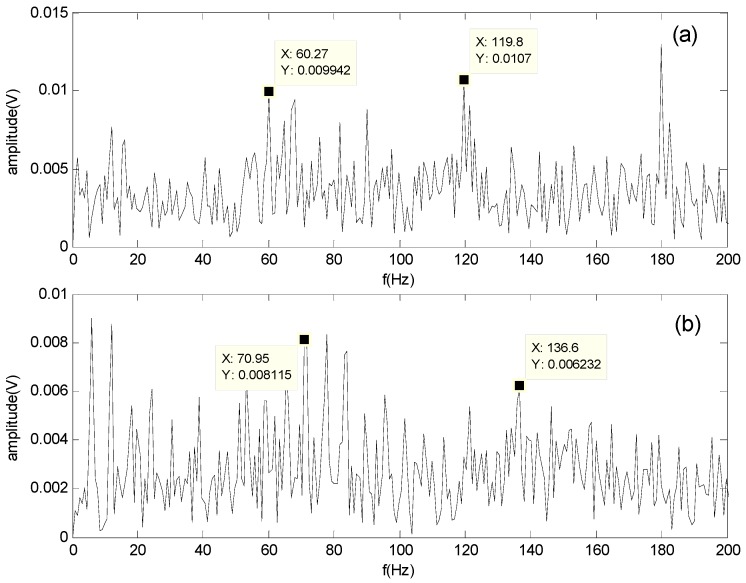
Spectrum of the separated noisy signals by the proposed method: (**a**) Spectrum of the outer-race defect; (**b**) Spectrum of the roller defect.

Then, the EEMD-ICA method and the proposed method are employed separately. As can be seen in Figure 14, the outer-race defect can be identified by the EEMD-ICA method; however, this method is not very effective for the roller defects. In contrast, Figure 15 shows that the roller defect, as well as the outer-race defect can be identified. Therefore, we can conclude that the proposed method is more effective than the EEMD-ICA method in the diagnosis of fault signals with low SNRs.

Next, we consider the operating time of the two methods. The two methods are made to run on the same computer. The relationships between the sampling points and the operating time of the two methods are listed in Table 2. As seen in the table, the EEMD method requires more operating time than the proposed method, and this time will increase with an increase in the number of sampling points.

## 7. Conclusions

In this paper, a new method that combines VMD with FastICA is proposed to extract the compound fault features of roller bearings. The study results show that the proposed method is more effective at separating the bearing outer-race defect and roller defect than the traditional envelope spectrum analysis. Furthermore, a comparison experiment shows that the proposed method has higher adaptability and practicability in separating strong noise signals than the ensemble empirical mode decomposition method. However, the proposed method needs much memory when this algorithm is operated on a computer, which is a drawback currently. Future work will focus on further optimization of the proposed method.

## Figures and Tables

**Table 1:** Fault characteristic frequencies of rolling bearing at different speeds.

Fault Characteristic Frequency			
	500 rpm	900 rpm	1300 rpm
Outer-race	33.2 Hz	59.8 Hz	86.3 Hz
rollers	39.3 Hz	71.8 Hz	102.3 Hz

**Table 2:** Operating time.

Operating Time			
Sampling Points	5000	10,000	500,000
EEMD-ICA method	12.8 s	27.6 s	150.7 s
The propose method	3.3 s	5.7 s	10.2 s
